# Two Rare Cases of Appendicitis: Amyand's Hernia and De Garengeot's Hernia

**DOI:** 10.1155/2019/6759206

**Published:** 2019-04-16

**Authors:** Kimberly A. Schaaf, Eric M. Melnychuk, Ross D. Ellison, Amy J. Snover

**Affiliations:** Department of Emergency Medicine, Geisinger Medical Center, 100 North Academy Avenue, Danville, Pennsylvania 17822, USA

## Abstract

An Amyand's hernia is an inguinal hernia that contains vermiform appendix. De Garengeot's hernias are similar; however, in this case the appendix is within a femoral hernia. Both types of hernia are rare, and those hernias associated with appendicitis, perforation, or abscess are even scarcer presentations. The treatment of Amyand's hernia and De Garengeot's hernia is not standardized. Generally, hernia repair is performed but disagreement remains regarding the use of mesh and performing appendectomy. This case series describes two individuals with appendicitis presenting to one emergency department within a 24-hour time frame. One case is of a patient with Amyand's hernia and another case is a patient with De Garengeot's hernia with an adjacent abscess. Both individuals were managed with appendectomy and hernia repair without the use of mesh.

## 1. Introduction

Hernias are at times complicated by the protrusion of abdominal contents within its lumen. Two very rare but similar entities are Amyand's hernia, described as a vermiform appendix within an inguinal hernia, and De Garengeot's hernia, being a vermiform appendix inside a femoral hernia [1–10]. Older studies claim that Amyand's hernia occurs in 1% of all inguinal hernias, and the presence of appendicitis within an Amyand's hernia accounts for 0.1% of all appendicitis [1–5, 7]. More recent research suggests the prevalence is smaller than previous thought, occurring in 0.4-0.6% of all inguinal hernias [1]. The prevalence of De Garengeot's hernias is even smaller, comprising only 0.5-5% of all femoral hernias. This is likely because femoral hernias occur much less frequently [8, 10]. The following cases of appendicitis were seen within hours at one emergency department (ED). To observe appendicitis within an Amyand hernia and De Garengeot's hernia in two patients at the same location within a short period of time is very unusual to say the least.

## 2. Case 1

An 82-year-old female with a history of asthma, gastroesophageal reflux disease, diverticulitis, ulcerative colitis, prior left hip replacement, and cholecystectomy presented to the ED with a 3-day history of right lower quadrant pain with associated nausea, nonbloody vomiting, and diarrhea. She was also complaining of a cough and back pain at the time of evaluation. She admitted to having a fall 3 weeks prior. Further review of systems was negative.

**Figure 1:**
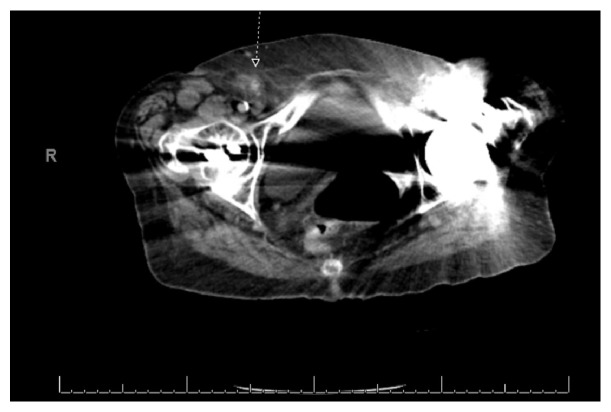
CT abdomen/pelvis image of Amyand's hernia is referenced by arrow.

**Figure 2:**
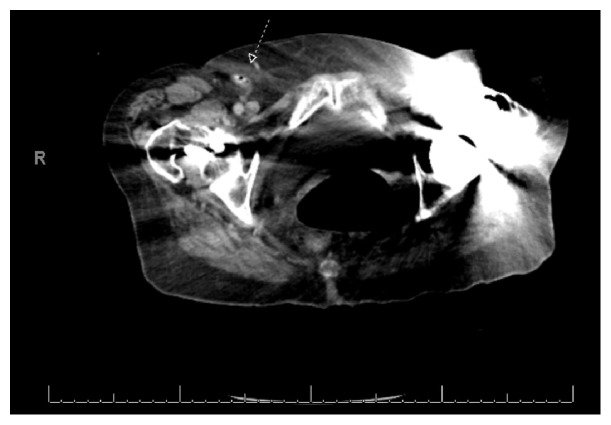
A second CT abdomen/pelvis image of Amyand's hernia is indicated by arrow.

Vital signs were blood pressure of 155/80 mmHg, pulse of 74 beats per minute, respirations of 18, and temperature of 36.7°C. Examination revealed a soft abdomen with right lower quadrant tenderness to palpation without evidence of an inguinal mass or erythema. Lab analysis was essentially normal. There was no leukocytosis. A CT scan of her abdomen was obtained due to her back pain and RLQ pain. The CT was interpreted by radiology as a right femoral hernia containing an inflamed appendix. Refer to Figures 1 and 2 for CT images.

The patient was treated operatively with laparoscopic appendectomy and by McVay hernia repair. No mesh was used during the repair of the hernia. The postoperative diagnoses were more complicated than what was visualized by radiology on the CT and included a Pantaloon hernia, a femoral hernia, and an Amyand's hernia containing an early, nonperforated appendicitis. The patient had no intraoperative or postoperative complications with the exception of pain, classified as Clavien-Dindo grade 1. On postoperative day 2, she was discharged to the skilled nursing facility where she resided. The patient's Charlson Comorbidity Index was calculated and her 10-year survival was estimated to be 53%.

## 3. Case 2

A 93-year-old female with a history of left ventricular hypertrophy, atrial fibrillation, hypertension, and no prior abdominal surgeries presented to the emergency department with dull, constant right lower quadrant pain for the past week. She saw her primary care physician who ordered an outpatient CT for possible hernia. The CT was concerning for appendicitis with adjacent abscess and hernia, so the patient was referred to the ED for further management. In the ED, she admitted to subjective fevers and melena at home. Review of systems revealed no other symptoms.

**Figure 3:**
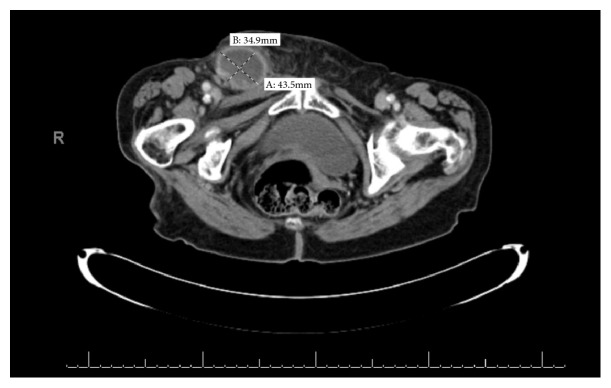
CT abdomen/pelvis image of right sided De Garengeot's hernia with adjacent abscess measuring 4.3 cm x 3.5 cm transversely.

Vital signs were blood pressure of 119/47 mmHg, pulse of 91 beats per minute, respirations of 16 per minute, temperature of 36.4°C, and oxygen saturation of 96% on room air. Examination revealed a soft abdomen with right lower quadrant tenderness to palpation and a nonreducible, erythematous groin mass. Lab analysis revealed a leukocytosis of 12.7 K/uL with a predominance of neutrophils. Radiology interpretation of the outpatient CT showed a right inguinal hernia containing vermiform appendix with adjacent abscess measuring 4.3 cm x 3.5 cm transversely. Refer to Figure 3 for CT imaging.

The patient underwent surgical management with appendectomy and McVay hernia repair. The appendix and adjacent abscess were accessed by way of the groin through the hernia. Intraoperatively, the hernia was found to be below the inguinal ligament in the femoral space. The abscess was drained, and the appendix was removed. The hernia was repaired without the use of mesh. The postoperative diagnosis was De Garengeot's hernia. The patient underwent no complications in the operating room or postoperatively with the exception of pain, Clavien-Dindo classification grade 1. She was discharged to home on postoperative day 3. Using the Charlson Comorbidity Index, her 10-year survival was estimated to be 21%.

## 4. Discussion

The individuals in this case series underwent management consistent with the approach agreed upon by most surgeons. Case 1, after surgical intervention, was found to have a type 2 Amyand's hernia for which laparoscopic appendectomy and McVay hernia repair without the use of mesh were performed. De Garengeot's hernia in Case 2 was complicated with an adjacent abscess. Surgical access was limited to the groin. The abscess and appendix were drained and removed by way of the hernia. The hernia was repaired by McVay repair without the use of mesh.

There is no standardized treatment for both Amyand's hernias and De Garengeot's hernias; however, similar management and concerns may be applied to both types of hernias [1–10]. There exists some controversy over the management of prophylactic appendectomy and whether mesh should be used during hernia repair for Amyand's hernias and De Garengeot's hernias. In addition, many cases may require individualized care that takes in regard comorbidities [1, 3]. This debate led Losanoff and Basson to propose a classification system with management principles to be applied to most cases of Amyand's hernia [1, 3, 4].

According to Losanoff and Basson, Amyand's hernias are categorized into four subtypes: (1) normal appendix within the inguinal hernia, (2) hernia with inflamed appendicitis, (3) hernia with perforation of the appendicitis, and (4) complications including abscess or malignancy [1, 3, 4, 7].

In subtype 1, Losanoff and Basson suggest Amyand's hernia may be managed with reduction or appendectomy, depending on comorbidities, and mesh hernioplasty [1, 3, 4, 7]. Subtypes 2-4, all with abnormalities of the appendix, require appendectomy and hernia repair without the use of mesh. Removal of the appendix may be performed by entrance through the hernia in cases of uncomplicated appendicitis, while laparoscopic appendectomy should be used in those complicated by abscess, perforation, or malignancy [1, 4].

As stated previously, there exists controversy over prophylactic appendectomy and the use of mesh [1–5]. In cases of Amyand's hernia with appendicitis or perforation, appendectomy should be performed. In those in which the appendix is normal without any inflammation, most believe appendectomy is not necessary. The appendix may be reduced, and the hernia is repaired with mesh. There are some that advocate appendectomy in all cases of Amyand's hernia [1, 2, 5]. These individuals generally believe that the appendix is prone to relocate within the hernia, and manipulation of the appendix during reduction may lead to appendicitis.

In all cases of uninflamed Amyand's hernia, hernia repair with mesh is acceptable [1–3, 5]. In regard to the debate of hernia repair with mesh, many believe that using mesh in the presence of inflammation or abscess increases the risk of wound infection, sepsis, fistula formation, and recurrent hernias. Nevertheless, there are authors that have used newer biologic meshes in cases of inflamed and perforated appendicitis without any development of infection [1, 4].

Management of De Garengeot's hernia is similar to Amyand's hernia. Because there are fewer cases of De Garengeot's hernia, there is no standardized treatment or consensus on management [8, 10]. It is generally believed that appendectomy should only be performed in cases of strangulation or inflammation, and mesh should not be used in the presence of inflammation. Again, controversy exists concerning prophylactic appendectomy and hernia repair with mesh. In appendicitis with abscess or perforation, the surgical approach should be restricted to the groin to avoid dissemination of infection to the abdomen [10].

The definitive treatment of Amyand's and De Garengeot's hernias is primarily the responsibility of the surgeon. The role of the emergency physician is to identify this rare presentation. Both patients in this case presented with typical symptoms of appendicitis and hernia, which prompted obtaining CT imaging. The ability to identify these variants on imaging is important and can be very challenging. In both cases, the location of the appendix and hernias were poorly visualized on CT imaging and were better identified in the operating room. The CT images for these patients are displayed in Figures 1–3.

In conclusion, Amyand's hernia and De Garengeot's hernia are extremely rare. The occasion to observe both in such a short time frame is unremarkable. The patients in this case series were managed following current recommendations of the majority, and they recovered without any significant complications.
